# Upper Airways Microbiota in Antibiotic-Naïve Wheezing and Healthy Infants from the Tropics of Rural Ecuador

**DOI:** 10.1371/journal.pone.0046803

**Published:** 2012-10-05

**Authors:** Paul Andres Cardenas, Philip J. Cooper, Michael J. Cox, Martha Chico, Carlos Arias, Miriam F. Moffatt, William Osmond Cookson

**Affiliations:** 1 National Heart & Lung Institute, Imperial College London, London, United Kingdom; 2 Fundación Ecuatoriana para la Investigación en Salud FEPIS, Quinindé, Esmeraldas Province, Ecuador; 3 Institute of Microbiology, Universidad San Francisco de Quito, Quito, Ecuador; 4 Colegio de Ciencias de la Salud, Universidad San Francisco de Quito, Quito, Ecuador; 5 Liverpool School of Tropical Medicine, Liverpool, United Kingdom; Ludwig-Maximilians-University Munich, Germany

## Abstract

**Background:**

Observations that the airway microbiome is disturbed in asthma may be confounded by the widespread use of antibiotics and inhaled steroids. We have therefore examined the oropharyngeal microbiome in early onset wheezing infants from a rural area of tropical Ecuador where antibiotic usage is minimal and glucocorticoid usage is absent.

**Materials and Methods:**

We performed pyrosequencing of amplicons of the polymorphic bacterial 16S rRNA gene from oropharyngeal samples from 24 infants with non-infectious early onset wheezing and 24 healthy controls (average age 10.2 months). We analyzed microbial community structure and differences between cases and controls by QIIME software.

**Results:**

We obtained 76,627 high quality sequences classified into 182 operational taxonomic units (OTUs). Firmicutes was the most common and diverse phylum (71.22% of sequences) with *Streptococcus* being the most common genus (49.72%). Known pathogens were found significantly more often in cases of infantile wheeze compared to controls, exemplified by *Haemophilus* spp. (OR = 2.12, 95% Confidence Interval (CI) 1.82–2.47; *P* = 5.46×10^−23^) and *Staphylococcus* spp. (OR = 124.1, 95%CI 59.0–261.2; *P* = 1.87×10^−241^). Other OTUs were less common in cases than controls, notably *Veillonella* spp. (OR = 0.59, 95%CI = 0.56–0.62; *P* = 8.06×10^−86^).

**Discussion:**

The airway microbiota appeared to contain many more Streptococci than found in Western Europe and the USA. Comparisons between healthy and wheezing infants revealed a significant difference in several bacterial phylotypes that were not confounded by antibiotics or use of inhaled steroids. The increased prevalence of pathogens such as *Haemophilus* and *Staphylococcus* spp. in cases may contribute to wheezing illnesses in this age group.

## Introduction

Asthma is a chronic disease of the airways that is characterized by an abnormal mucosa, intermittent airway inflammation and symptoms of wheezing, dyspnea and cough. The syndrome results from a complex interplay between genetic and environmental factors [1].

A worthwhile understanding of the causes of asthma needs to reconcile consistent epidemiological indications of the importance of the microbiome (also known as microbiota) to the disease [1]. These include the protection afforded by a rich microbial environment in early life [2], [3], observations that the bronchial tree contains a characteristic flora that is disturbed by the presence of pathogens such as *Haemophilus infuenzae* in asthma [4], [5], birth cohort studies showing that the presence of the same pathogens in throat swabs predicts the later development of asthma [6] and recognition that these bacteria have consistently been associated with exacerbations of asthma [7].

Of potential importance is the finding that organisms commonly found in healthy airways and mucosal surfaces are significantly reduced in asthmatic airways [4]. Investigations of inflammatory bowel disease have shown that a normal bacterial flora is essential in maintaining a healthy mucosa [8] and similar mechanisms are likely to be important in the airways [9]–[11]. Murine studies have shown that sterility of the airways and intestinal tract results in enhanced inflammatory responses to a variety of stimuli [10], [12].

Ninety percent of the cells in the human body are microorganisms including bacteria, parasites and archaea [13]. These microorganisms are commensal on body surfaces exposed to the external environment including the gut, respiratory tract and skin. Allergy, and other immune diseases are associated with differences in microbial communities, but it is unclear if these differences are causes or consequences of disease [4], [5], [12]. Although most bacteria are not cultivable with standard methods [14], the membership of complex microbial communities can be quantified and classified by DNA sequencing of the conserved bacterial 16S rRNA gene [15], [16]. Bacteria are classified by these sequences into Operational Taxonomic Units (OTUs). OTUs approximate closely but not completely to taxonomy derived from classical techniques, and sequences of other regions may be necessary for precise discrimination at the species level.

Previous studies of the airway microbiome in healthy and in diseased subjects have been carried out in Westernized societies [4]–[6] where antibiotic use and the prescription of inhaled corticosteroids is almost ubiquitous and confounds understanding of the microbiome. We have therefore carried out a sequence-based study of the upper airway microbiome in children from the Esmeraldas province in rural Ecuador who have had minimal exposure to antibiotic medications and no exposure to inhaled steroids.

Ecuador has strong regional differences in the prevalence of wheeze in rural compared to urban areas. The prevalence of wheezing in children has been estimated to be 16.6% in urban areas [17] while in rural areas of the Pichincha province the rate of current wheezing has been estimated between 0.8% and 2.2% [18], [19]. Factors that may be protective against asthma in this region include exposures associated with living in a rural environment, low antibiotic usage and a high rate of geohelminth parasitic infections. We therefore sought to compare and contrast the airway microbiome in infants with non-infective wheeze and healthy controls, and to relate our findings to surveys of the airway microbiome in European children.

## Materials and Methods

### Subjects

A case-control study was designed to investigate the upper airway microbiota profiles of early onset non-infectious wheezing infants (cases) and healthy infants (controls). The project used DNA extracted from oropharyngeal swabs samples collected from the hypopharynx of 48 infants (average age 10.2 months) that were recruited as part of a birth cohort (the ECUAVIDA cohort) in the Esmeraldas Province in Ecuador. The aim of the ECUAVIDA cohort study is to investigate the effects of early infant infections on the development of immunity, allergic sensitization and allergic disease and the methodology has been previously described in detail [18], [19]. The study is an unselected population-based birth cohort that has recruited 2,403 newborns in the rural District of Quininde in the Esmeraldas Province, Ecuador. Detailed data has been collected from the mothers at the time of the first antenatal visit using questionnaires and environmental sampling. The protocol for the ECUAVIDA cohort was approved by the Ethical Committees of the Hospital Pedro Vicente Maldonado and Universidad San Francisco de Quito, Quito, Ecuador.

Twenty-four infants were selected with early onset multiple-trigger non-infectious wheezing according to the GINA guidelines (http://www.ginasthma.org/). Wheezing illness was diagnosed by a physician. Twenty-four healthy controls (no history of wheezing, current respiratory disease, chronic disease or current infections) were selected and paired by age range to cases. The samples were collected when the cases and controls did not have any evidence of a current airway infection (cold symptoms and fever). The infants in both groups had a minimal history of antibiotic use, with 87% never being exposed to antibiotics, and the whole group receiving on average 0.12 courses of antibiotics per child (Table 1). None of the infants had received antibiotics for any reason for at least two weeks prior to sampling. None of the subjects had ever received corticosteroids. All subjects were of the same mixed ethnic background, lived in the same town and had access to the same basic electricity, water, and sanitation services. All subjects had received vaccines recommended by the Ecuadorian Ministry of Public Health. None had received anti-Streptococcal vaccination.

**Table 1:** Epidemiologic characteristics of the children investigated in the study.

	Cases	Controls
Mean age (months)	9.9	10.5
Sex (% male)	50%	42%
Number of Individuals per room of the house	3.57	3.11
Average parental income (USD per month)	186	238
Average Birth weight (grams)	3306	3154
Maternal Education Level (percentage)		
Illiterate	0%	4%
Primary School Incomplete	21%	8%
Primary School Complete	17%	33%
High school Incomplete	42%	46%
High school Complete	21%	4%
University Incomplete	0%	4%
University Complete	0%	0%
Respiratory Tract Infections (average occurrences per month of age)	0.21	0.15
Average Number of Antibiotic Courses	0.166	0.083
Antibiotics (number of times used )		
Trimethoprim/sulfamethoxazole	1	
Cephalexine		1
Amoxicilin	3	1
Antibiotic Use for Respiratory Tract Infection		
Upper RTIs	1	1
Lower RTIs	1	1

### Sample Collection and Storage

Throat swabs were collected by a physician using sterile cotton swabs and were placed in collection tubes (Qiagen, UK). Sampling was performed carefully without touching any surface other than the oropharynx and using a tongue depressor. Each swab was rubbed approximately five times around the oropharynx. An even pressure was applied and the swab was rotated without interruption. Post sampling, the swab was immediately placed back into the collection tube and stored at −20°C and subsequently within the next 24 hours at −80°C. Samples were shipped to Imperial College, London on dry ice.

**Figure 1:**
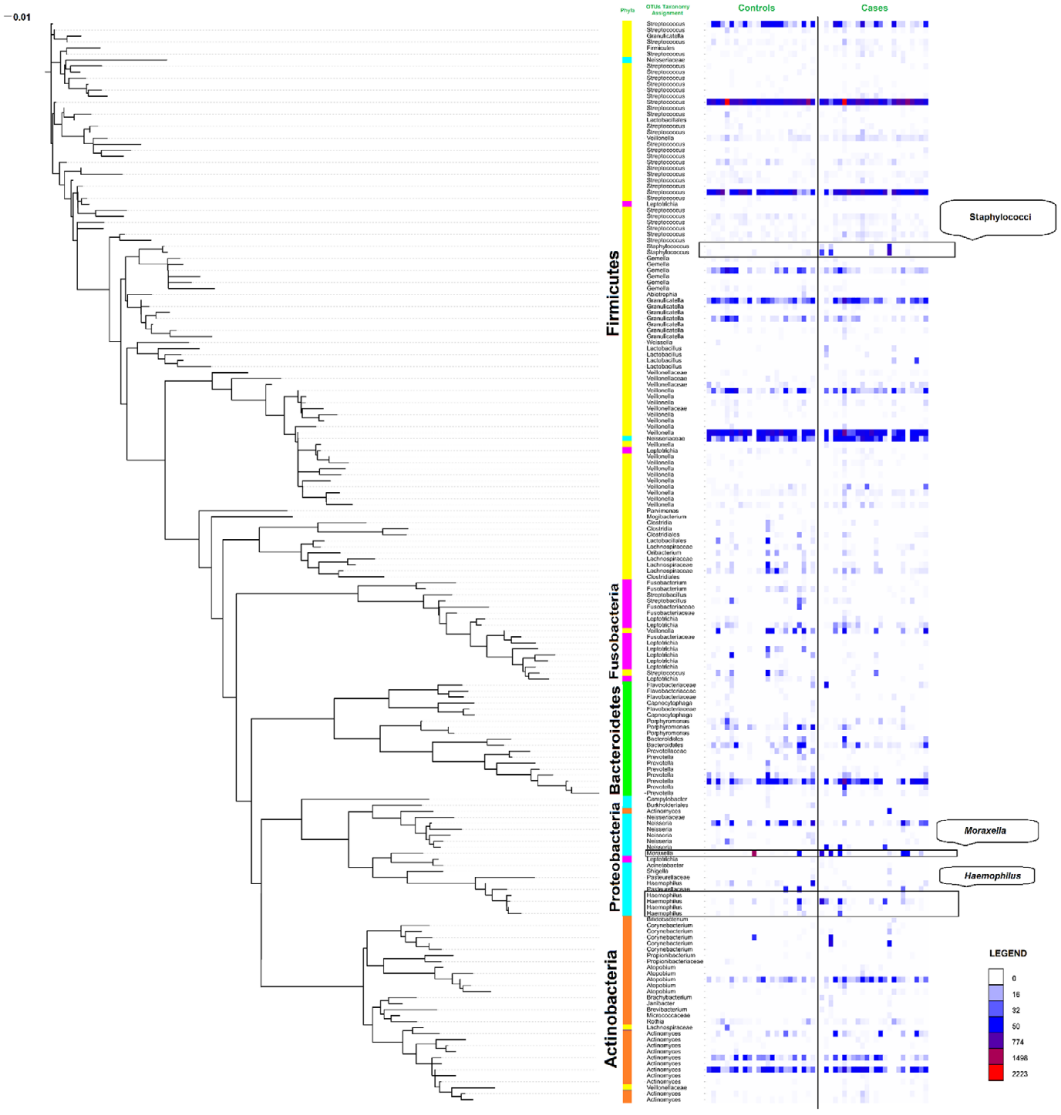
Phylogenetic tree and Heatmap of bacterial 16S rRNA sequences derived from throat swabs. Total sequence counts for individual operational taxonomic units (OTUs) are shown in the right column. Taxonomy assignments at the phylum level are shown in the inner column and colour coded. Intrusions of different colours within particular phyla indicate discrepancies between the phylogenetic and database classifications.

### Bacterial DNA Extraction from Throat Swabs

Bacterial DNA was extracted from the throat swabs using a modified protocol of the commercial QIAmp DNA Mini Kit (Qiagen). Additional steps at the beginning of the protocol were included to improve the lysis of Gram positive bacteria [4].

Each swab head was transferred into a 2 ml microcentrifuge tube, and 432 µl TE (Tris EDTA pH 8.0)+18 µl 4X lysozyme solution was added (4X lysozyme in a concentration of 1000 U/µl was prepared from lysozyme stock at 30,000 U/µl Ready-Lyse™ Lysozyme Solution of EPICENTRE, UK).

Samples were incubated for 1 hour at 37°C; to allow improved lysis of Gram +ve bacterial walls. During this hour, samples were vortexed for 20 seconds at intervals of 15 minutes. Next 30 µl of Proteinase K and 450 µl of Buffer AL were added to the tube and samples were incubated at 56°C for 30 minutes. To terminate the Proteinase K step, samples were then incubated for 5 min at 95°C.450 µl of Ethanol (96–100%) was added to the sample. In order to obtain a homogenous solution this was mixed by vortexing. This solution was then applied to the QIAamp Spin Column as per the manufacturer’s protocol. In the final step 40 µl of nuclease free water was added instead of the elution buffer supplied by the kit. If the DNA was not being used immediately after extraction samples were stored at −20°C until required.

**Figure 2:**
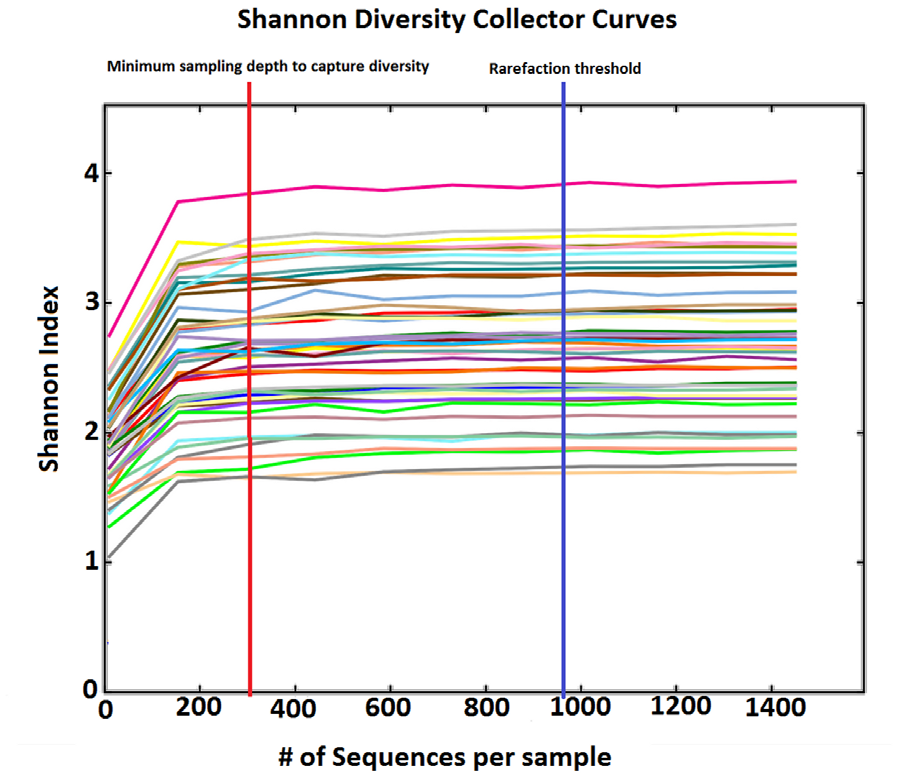
Shannon diversity collector curves. Multiple rarefaction curves were collated from each sample’s Shannon diversity index. The graphic shows the estimated diversity plotted against the number of sequences per sample. Each line represents one sample. The plateau in each estimated diversity curve indicates the minimum number of sequences to capture diversity. For all samples the plateau was achieved at approximately 320 sequences (red vertical line), well below our chosen rarefaction threshold of 969 sequences (blue line).

### Amplification of 16S rRNA Gene

Polymerase chain reaction (PCR) was used to amplify the variable regions 3 to 5 (V3–V5, Primers 454B_357F and 454A_926R [20]) of the gene that encodes for 16S rRNA in bacteria. Samples were multiplexed using sample specific barcodes [20] and Roche 454 adaptor sequences (Roche Diagnostics, Oakland). To minimize PCR nucleotide insertion mistakes, a high fidelity *Taq* polymerase was used, and samples were amplified in quadruplicate reactions with 20 cycles each and then pooled.

**Table 2:** Differences in bacterial 16S rRNA sequences from throat swabs of infants in rural Ecuador with non-infectious wheeze and healthy controls.

Groups*	Number of Sequences		Number of subjects (%) in which OTU groups were detected at >1%		*P* value	Odds Ratio	95% CI
	Controls	Cases	Controls	Cases			
**Actinobacteria/Actinomyces**	1192	1306	23 (96%)	21 (88%)	1.89×10^−02^	1.10	1.02 to 1.20
**Actinobacteria/Atopobium**	173	388	19 (79%)	20 (83%)	8.99×10^−20^	2.27	1.89 to 2.71
**Actinobacteria/Corynebacterium**	27	656	6 (25%)	7 (29%)	1.37×10^−129^	25.0	17.0 to 36.7
**Bacteroidetes/Bacteroidales**	227	126	18 (75%)	18 (75%)	9.57×10^−08^	0.55	0.44 to 0.69
**Bacteroidetes/Flavobacteriaceae**	19	167	7 (29%)	8 (33%)	4.02×10^−31^	12.1	7.55 to 19.3
**Bacteroidetes/Porphyromonas**	264	53	19 (79%)	16 (67%)	2.81×10^−32^	0.20	0.15 to 0.27
**Bacteroidetes/Prevotella**	930	1263	23 (96%)	20 (83%)	3.24×10^−13^	1.38	1.27 to 1.50
**Firmicutes/Gemella**	353	143	19 (79%)	22 (92%)	4.29×10^−21^	0.40	0.33 to 0.49
**Firmicutes/Lachnospiraceae**	209	81	18 (75%)	15 (62%)	7.79×10^−14^	0.39	0.30 to 0.50
**Firmicutes/Staphylococcus**	7	837	6 (25%)	5 (21%)	1.87×10^−241^	124.1	59.0 to 261.2
**Firmicutes/Veillonella**	4117	2623	23 (96%)	23 (96%)	8.06×10^−86^	0.59	0.56 to 0.62
**Fusobacteria/Leptotrichia**	236	99	17 (71%)	17 (71%)	9.37×10^−14^	0.42	0.33 to 0.53
**Proteobacteria/Haemophilus**	248	520	14 (58%)	17 (71%)	5.46×10^−23^	2.12	1.82 to 2.47
**Proteobacteria/Moraxella**	931	745	5 (21%)	9 (38%)	4.54×10^−06^	0.79	0.72 to 0.88
**Proteobacteria/Neisseriaceae**	1032	1218	16 (67%)	18 (75%)	5.84×10^−05^	1.19	1.09 to 1.30
**Proteobacteria/Pasteurellaceae**	163	32	4 (17%)	4 (17%)	1.13×10^−20^	0.20	0.13 to 0.29

* Results are shown for the lowest level of taxonomic identification achieved. Only groups with more than 100 sequences and statistically significant differences are shown. *P* values are corrected for multiple comparisons.

### Pyrosequencing and Data Analysis

The DNA amplicons were pyrosequenced using a GS Junior Titanium 454 (Roche Diagnostics, Oakland) following manufacturer’s protocols (http://www.gsjunior.com/454-gs-junior.php). Data analysis was performed using the software “Quantitative Insights into Microbial Ecology” (QIIME) [21]. Reads were removed if they were <200 and >800 nucleotides (nt) in length, if there were mismatches in the barcodes or primers, if ambiguous nucleotides were present or if the read quality score was <25. The denoiser algorithm version 1.2.1 [22] was used to avoid overestimation of diversity and chimeras were removed using ChimeraSlayer [23]. Phylogenetic classification was assigned using the Ribosomal Database Project database (RDP). [24] Sequences were clustered in Operational Taxonomic Units (OTUs) using UCLUST version 2.1 at 97% sequence identity [25]. Any sequences present once (singletons) or in only one sample were filtered out.

**Figure 3:**
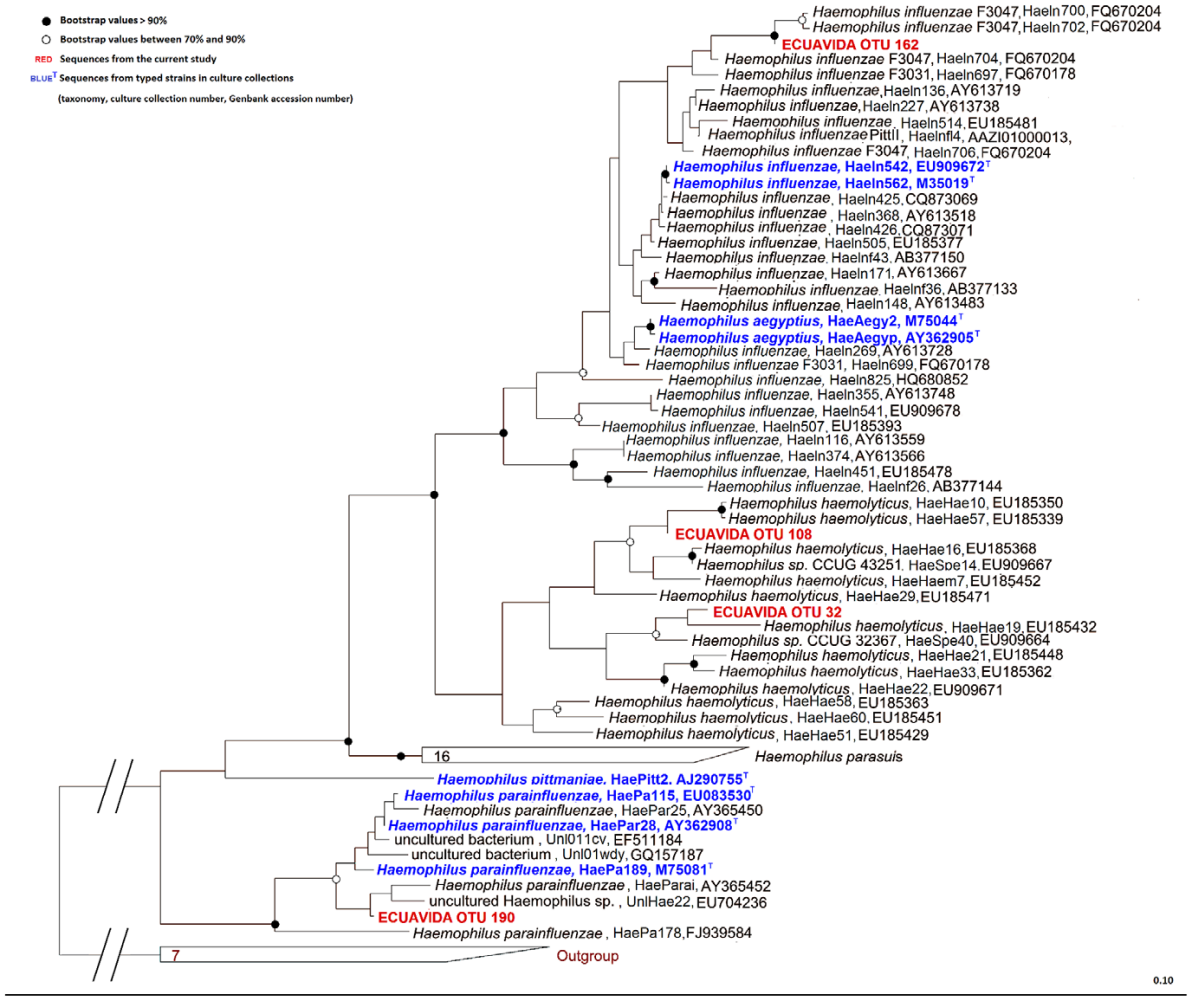
Phylogenetic identification of *Haemophilus* OTUs. Phylogenetic analysis of the 16S rRNA sequences of the OTUs assigned taxonomically to *Haemophilus* genus (OTUs 32, 108, 162 and 190, shown in Red) together with reference *Haemophilus* sequences from the SILVA database, using the ARB alignment editor. The scale bar indicates 10% sequence divergence, and NCBI accession numbers are included. The tree was rooted with a near neighbour outgroup constructed with sequences from *Morganella morganii*, *Proteus mirabilis* and *Providencia stuartii*.

Remaining sequences were aligned using PyNast [26]. Sequences were rarefied (to remove the heterogeneity of the number of sequences per sample) prior to calculation of alpha and beta diversity statistics. Alpha diversity indexes were calculated in QIIME from rarefied samples using for diversity the Shannon index [27], for richness the Chao1 index [28], and evenness by the equitability index [29]. Beta diversity was calculated using weighted and unweighted UniFrac [30] and principal coordinate analysis (PCoA) performed. Neighbour joining with nearest neighbour interchange phylogenetic trees were created using the representative sequences of each OTU and FastTree version 2.1.3 [31]. The heatmap to represent the abundance of sequences was constructed in iTOL [32]. Microbial community comparisons were performed using parametric statistics in METASTATS, with the *P* values corrected by multiple hypothesis testing using the false discovery rate (FDR) [20]. Sequences are available in EBI (European Bioinformatics Institute) with the accession number ERP001558.

**Figure 4:**
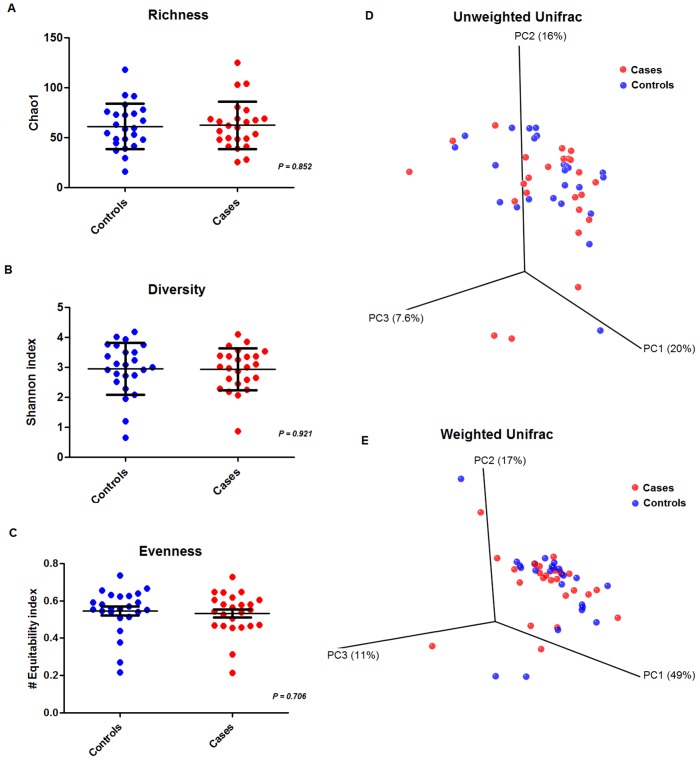
Alpha and Beta diversity comparisons between Cases and Controls. A) Scatter dot plot comparing cases versus controls values of chao1 richness index. B) Scatter dot plot comparing values of Shannon diversity index. C) Scatter dot plot comparing equitability evenness index. D) Unweighted UNIFRAC Principal Coordinate Analysis (PCoA) plot comparing presence/absence metrics. E) Weighted UNIFRAC Principal Coordinate Analysis (PCoA) plot comparing presence/absence metrics and abundance.

Representative sequences from significantly different OTUs of further interest were investigated using more intensive phylogenetic approaches in order to maximize the quality of the identification. These test sequences were aligned using the online SINA aligner (http://www.arb-silva.de/aligner/version 1.29 [33]) and this was imported into the ARB phylogenetic software (version 5.1, http://www.arb-home.de/ [34]) running on Biolinux 6.0 (http://nebc.nerc.ac.uk/tools/bio-linux/bio-linux-6.0, ref 3 [35]). The aligned SILVA reference database SSU_REF108 of 618,442 high quality 16S rRNA gene sequences was downloaded and merged with the aligned test sequences. All *Haemophilus* spp. (or *Streptococcus* spp.) sequences within the database were selected and the SINA alignment individually checked for each test sequence in the ARB alignment editor. The length of the alignments used depended on the length of the available reference reads for each OTU. Thus, for the *Haemophilus* spp. alignment, the 522 bp region corresponding to the region between positions 384 and 908 of the *Escherichia coli* reference were selected, and for *Streptococcus* spp. 470 bp between positions 470 and 908. Columns of the alignment containing uninformative positions (gaps) were masked from the phylogenetic analysis. Three trees were constructed for each of the genera, an ARB neighbor joining (NJ) tree with 1000 bootstraps, a Maximum Parsimony (MP) tree with 500 bootstraps and a RAxML Maximum Likelihood (ML) tree (version 7.0.3, [36]) with GTR substitution model in rapid hill-climbing mode. Trees were rooted with sequences from near neighbours outside the genus of interest. Tree topology was compared between the three methods and bootstrap values for the NJ and MP trees were used to determine stability of the phylogeny. Accession numbers for the reference sequences used are recorded in the tree labels and those for the outgroup were DQ358146, AJ301681, AF008582, AJ301682, AF008581, AM040491, and AM040495.

## Results

Examination of the clinical data showed no significant differences between the 24 cases of non-infectious wheezing and the 24 controls in age, sex and antibiotic use (Table 1). The controls appeared to have higher parental income ($238 per month compared to $186) and slightly fewer individuals per room in their houses (3.1 compared to 3.6), although only 4% of their mothers had finished high school compared to 21% of cases.

An initial 108,042 raw sequences were obtained from all subjects. After denoising, singleton exclusion, chimera checking and removal of OTUs present in only one sample a total of 76,627 sequences remained (37,235 in cases and 39,392 in controls). Between 969 and 6269 sequences were obtained per sample. In order to control sample heterogeneity, sequences were rarified to the same minimum of 969 for all subjects. Consequently, we identified 182 operational taxonomic units (OTUs) at 97% sequence identity level that were assembled into a phylogenetic tree using FastTree and iTOL (Figure 1).

By extrapolation of collectors’ curves we estimated that the samples contained an average of 289.8 OTUs (95% CI 218.39–361.30). Multiple rarefaction curves using the Shannon index (Figure 2) showed that a plateau of diversity was achieved in around 360 sequences per sample. This value would be considered as the minimum sampling depth to capture diversity. Our rarefaction was performed at a minimum of 969 sequences per sample, which therefore is a realistic panorama of each sample’s diversity. Phylogenetic classification of the ungrouped sequences showed a high prevalence of the phylum Firmicutes (72% of the total number of sequences obtained) followed by Proteobacteria (12%), Actinobacteria (8%), Bacteroidetes (7%) and Fusobacteria (1%) (Figure 1). Firmicutes was the most diverse phylum containing 93 distinct OTUs (51% of the total OTUs), followed by Actinobacteria with 30 OTUs (16%), Proteobacteria with 20 OTUs (11%), then Fusobacteria and Bacteroidetes with 18 and 19 OTUs respectively (10%). *Streptococcus* was the most common genus (49.72% of the total) followed by *Veillonella* (14.5%), *Atopobium* (5.37%) and *Prevotella* (4.72%).

We performed analysis of OTU taxonomy assignments summarized at genus level (when it was not possible to define at genus level, the next best determined taxonomy assignment was used), and the number of samples from where differences were detected at greater than 1% was included (Table 2). We found that members of the groups *Actinomyces* (*P* = 1.89×10^−02^, OR 1.10), *Atopobium* (*P* = 8.99×10^−20^, OR 2.27), *Corynebacterium* (*P = *1.37×10^−129^, OR 24.99), *Flavobacteriaceae* (*P* = 4.02×10^−31^, OR 12.07), *Prevotella* (*P* = 3.24×10^−13^, OR 1.38), *Staphylococcus* (*P* = 1.87×10^−241^, OR 124.11), *Neisseriaceae* (*P* = 5.84×10^−05^, OR 1.19), and *Haemophilus* (*P* = 5.46×10^−23^, OR 2.12) occurred highly significantly more often in the cases of infantile wheeze compared to non-wheezing controls.

By contrast in controls there was a significantly higher prevalence of *Bacteroidales* (*P* = 9.57×10^−08^, OR 0.55), *Porphyromonas* (*P* = 2.81×10^−32^, OR 0.20), *Gemella* (*P* = 4.29×10^−21^, OR 0.40), *Lachnospiraceae* (*P* = 7.79×10^−14^, OR 0.39), *Veillonella* (*P = *8.06×10^−86^, OR 0.59), *Leptotrichia* (*P = *9.37×10^−14^, OR 0.42), *Pasteurellaceae* (*P = *1.13×10^−20^, OR 0.20) and *Moraxella* (*P = *4.54×10^−06^, OR 0.79) (Table 2). We repeated the statistical analysis excluding the 6 children that had a minimal use of antibiotics and obtained similar results to the full sample set.

Inspection of the data for individual subjects (Figure 1) showed that the high abundance of *Moraxella* sp. in a single sample resulted in the whole genus being significantly more prevalent in controls. If this particular sample was excluded from the analysis, *Moraxella* became more prevalent in cases.

The biological interpretation of the differences in the frequencies of individual OTUs is limited by the imprecision of OTU assignments in identifying individual species. In particular, this problem was obvious in our data with OTUs assigned to *Streptococcus* and *Haemophilus spp.*, which were likely to contain a mixture of pathogenic and non-pathogenic strains. We therefore attempted to improve the classification of these OTUs by including them in phylogenetic tress constructed from reference sequences.

Using three independent phylogenetic treeing methods it was not possible to increase the specificity of the identification of the *Streptococcus* spp. OTUs beyond that of the basic Ribosomal Database Project (RDP) classifier. Tree topology between the three methods of treeing was not conserved and significance of assignment to major nodes was low.

By contrast, the tree topology for *Haemophilus* spp. was conserved in all three methods of phylogenetic inference (Figure 3), with robust significance for assignment for each *Haemophilus* spp. OTU. This enabled confident assignment of OTU 162 to *Haemophilus influenzae*, OTU 32 and 38 to *Haemophilus haemolyticus,* and OTU 190 to *Haemophilus parainfluenzae*. OTU 162 which was assigned to the known pathogen *Haemophilus influenza*e was significantly more common in cases (*P* = 1.66×10^−49^, OR 3.45) compared with controls, whilst OTU 190 (assigned to *Haemophilus parainfluenza*e) was significantly more prevalent in controls (*P* = 2.74×10^−48^, OR 0.05).

We next tested if changes in the abundance of individual OTUs were associated with alterations in the overall structure of the microbial populations between cases and controls. We did not detect any differences between cases and controls in species richness, taxa abundances, or evenness (Figure 4 A, B and C). We found no large-scale differences in microbial community cluster patterns between cases and controls (beta diversity) using the principal coordinate analysis (PCoA) of the UniFrac distance matrix (weighted and un-weighted) (Figure 4, D and E).

## Discussion

This study has shown significant perturbations of the airways microbiota in infants with early onset non-infectious wheeze from a rural district in the tropics of Ecuador. Previous studies conducted in a geographically adjacent area with similar climatic, population, and geographic characteristics estimated the prevalence of asthma in school age children to be just 2.2% [18] and our subjects were characterized by minimal use of antibiotics and a complete absence of use of corticosteroids and anti-Streptococcal vaccination. Our samples may therefore exemplify a naturally developing microbial community in the early months of life. Bacterial diversity in these infants (estimated mean 290 OTUs) may be higher than that in seen previously in European children (mean 85 OTUs) [4], although differences in methodology mean that this inference should be treated with caution.

It is of interest that Firmicutes was the most common and most diverse phylum with *Streptococcus* the most prevalent genus, consistent with observations of the airways microbiota in European children [4]. We were not able to differentiate well between members of the important *Streptococcus* group on the basis of the 16S sequences, and the typing of alternative chronometers (such as *pheS*, *rpoA*, and *gyrB* ) and the incorporation of mutilocus sequence analysis (MLS) [37] will be important in future studies of the airway microbiome.

We defined our phenotype in infants by non-febrile episodic wheezing, according to the GINA guidelines (http://www.ginasthma.org/). These guidelines recognize that asthma diagnosis before the age of 6 years is complicated by the difficulty in performing accurate lung function tests. The recognition of recurrent wheezing not related to infections is as a consequence the most important diagnostic indicator of asthma in this age group [38]. Because not all children who experience wheezing events will develop asthma and not all children with asthma wheeze [39], we have followed the recommendation that the phenotype of recurrent non-infectious wheezing syndrome be applied to children less than 24 months old [38], [39].

We used culture-independent molecular techniques to characterize microbial communities and to quantitatively investigate dissimilarities. We, and others have shown previously that the microbiome of the oropharnyx correlates with that of the bronchial tree assessed by brushings or by lavage [4], [40], and therefore oropharyngeal swabs were used for this epidemiological survey. Pyrosequencing robustly determines the diversity and abundance of microbial communities in a quantitative and qualitative form. The assignment of approximately 1,600 individual sequences for each subject has conferred substantial statistical power to our study. We have reduced the possibility of bias by stringent removal of chimeric sequences, sequences present only once in the dataset and OTUs that were present in only one sample.

We were aware that a high abundance of sequences in few samples could drive the overall prevalence of bacteria in cases or controls, as occurred with *Moraxella* spp. in this study. We have therefore confined our tests for significant differences to OTUs present in three or more subjects with a total of more than 100 sequences per sample.

Our study identified a higher frequency of potential pathogens (*Neisseriaceae*, *Prevotella*, *Corynebacterium*, *Staphylococcus, Actinomyces* and *Haemophilus*) in wheezy infants compared to healthy controls. This finding is consistent with substantial epidemiological studies of European neonates that used standard bacterial cultures to show carriage of pathogens in neonates predicted later asthma risk [6]. The finding is also consistent with earlier studies in older children and adults with asthma that used 16S rRNA gene sequencing for bacterial characterisation [4].

The incorporation of reference strain sequences into our phylogenetic trees allowed us to discriminate between *Haemophilus* spp. OTUs at species level, and to show that the pathogen *H. influenzae* was more prevalent in wheezing infants, in concordance with previous studies [6]. *H. parainfluenzae* was more abundant in healthy children which might be associated with wheezing protection. The 16S rRNA gene has previously been suggested for use as a marker in MLST of *H. influenzae* [41] and our results confirm that its phylogeny is well matched to species and strain identification in airway samples.

In this study we found a lack of potentially ‘protective’ bacterial genera in non-infectious wheezing infants compared with controls, particularly *Veillonella*, *Pasteurellaceae* and *Gemella.* Alterations in the normal microbiota may alter the host resistance to pathogen colonization in the gut [42] and in the airways [43]. Possible mechanisms for this include direct inhibition of pathogen growth by commensal secreted factors [44]. Commensal bacteria may also elicit tonic signals in the gut epithelium that prevent activation of innate and adaptive immune responses [45], [46]. It may be relevant that manipulating the airway microbiome in gnotobiotic mice may produce major changes in airway responsiveness and immunity [10], [11].

Our results suggest that the upper airways microbiota of early onset wheezing infants from the tropics exhibit an increase in the frequency of pathogenic bacterial OTUs that is not confounded by antibiotic or steroid medications. These specific differences do not appear to have been accompanied by detectable community changes in the numbers of species and their overall relative distribution in our samples. Larger studies and direct measurements of the communities in the lower airways may change this perception. The results provide further support for a hypothesis that pathogenic bacteria may contribute to a wheezy diathesis in infants [6].
